# Supplementary material for: Sweet Cherry Plants Prioritize Their Response to Cope with Summer Drought, Overshadowing the Defense Response to Pseudomonas syringae pv. syringae
Source: Plants (Basel). 2024 Jun 24;13(13):1737. doi: 10.3390/plants13131737 (PMC11243571; doi:10.3390/plants13131737)

Supplemental Data

Supplemental Data S1.

Bacterial detection by qPCR at the Inoculation Site in branches at 13 dpi and leaves at 81 dpi.

DNA extraction

Plant samples from twigs or leaves were collected and ground through mechanical disruption using a mortar, pestle, and liquid nitrogen. For DNA extraction, the FavorPrep™ Plant Genomic DNA Extraction Mini Kit (Favorgen) was utilized. All steps were conducted in accordance with the manufacturer's protocol.

Quantification of Pss load in sweet cherry samples using qPCR.

Calibration curves for DNA quantification were generated through qPCR using seven 10-fold serial dilutions of DNA extracted from the *Pss*A1M3 strain cultivated on King Agar B. Additionally, total DNA was extracted from a leaf sample of sweet cherry cv. 'Lapins'. The qPCR reactions utilized Brilliant III SYBR® Green qPCR Master Mix (Agilent Technologies, CA, USA), following the provided instructions. These reactions were performed on an AriaMx Real-time PCR System. Each qPCR reaction consisted of 200 nM of each primer, 1 µL of the respective dilution, and Molecular Biology Grade Water (Corning®) to reach a final volume of 15 µL. For plant DNA extraction, a 20-fold dilution was applied using Molecular Biology Grade Water (Corning®) before conducting the qPCR reaction. To quantify sweet cherry DNA within each sample, specific primers for the *sar deficient 1* (*Pasard1*) gene were utilized (Supplementary Table). For quantifying *Pss* DNA, primers targeting the *Glutathione-dependent formaldehyde-activating enzyme 1* (*gfa*) gene were employed (Supplementary Table). All qPCR reactions were performed in duplicate at a minimum. Using the STATGRAPHICS Centurion XV software, the resulting calibration equation for sweet cherry was: log10 [sweet cherry DNA (ng/µL)] = (Cq – 20.7584)/(-2.50357), with a p-value = 0.0038 and R^2*100 of 99.243%. For *Pss*, the calibration equation was as follows: log10 [*Pss* DNA (ng/µL)] = (Cq - 13.6263)/(-3.302), with a p-value < 0.00001 and R^2*100 of 99.947%. The *Pss* load in each sample was determined by calculating the ratio between the quantification of *Pss* DNA and sweet cherry DNA. Positive controls included purified DNA from the *Pss*A1M3 strain and purified DNA from a non-inoculated *Prunus avium* twig.

Supplemental data S2

Microbiological analysis of bacterial Growth in sweet cherry branches 5 cm below to the inoculation site at 13 dpi.

Bacterial growth was studied in inoculated shoots after 13 days of inoculation with the *Pss* strain. The shoot was cut 5 cm below the inoculation site and then it was washed 3 times with sterile distilled water and divided into 1 cm sections. Each piece of tissue was placed in a conical tube with 10 ml of 0.8% KCl solution under agitation for 24 hours to isolate bacterial cells in the sample. A 1:10 dilution of each suspension was plated on PAF medium and petri dishes were incubated at 26 ºC for 24 hours. Each sample was analyzed in triplicate. *Pss* positive samples were confirmed by PCR detection of *syrB* and *syrD* genes.

Supplemental data S3

All the weather and agrometeorolical data were obtained from the INIA Rayentué Agrometeorological station (https://agrometeorologia.cl/), located approximately 500 m from the experimental site. Air temperature, relative humidity and ET_0 were obtained at 1h intervals.

Supplemental data S4. Pictures of sweet cherry plants cvs. Bing and Santina from the control (A,B,C) and *Pss* inoculation (D,E) treatments.

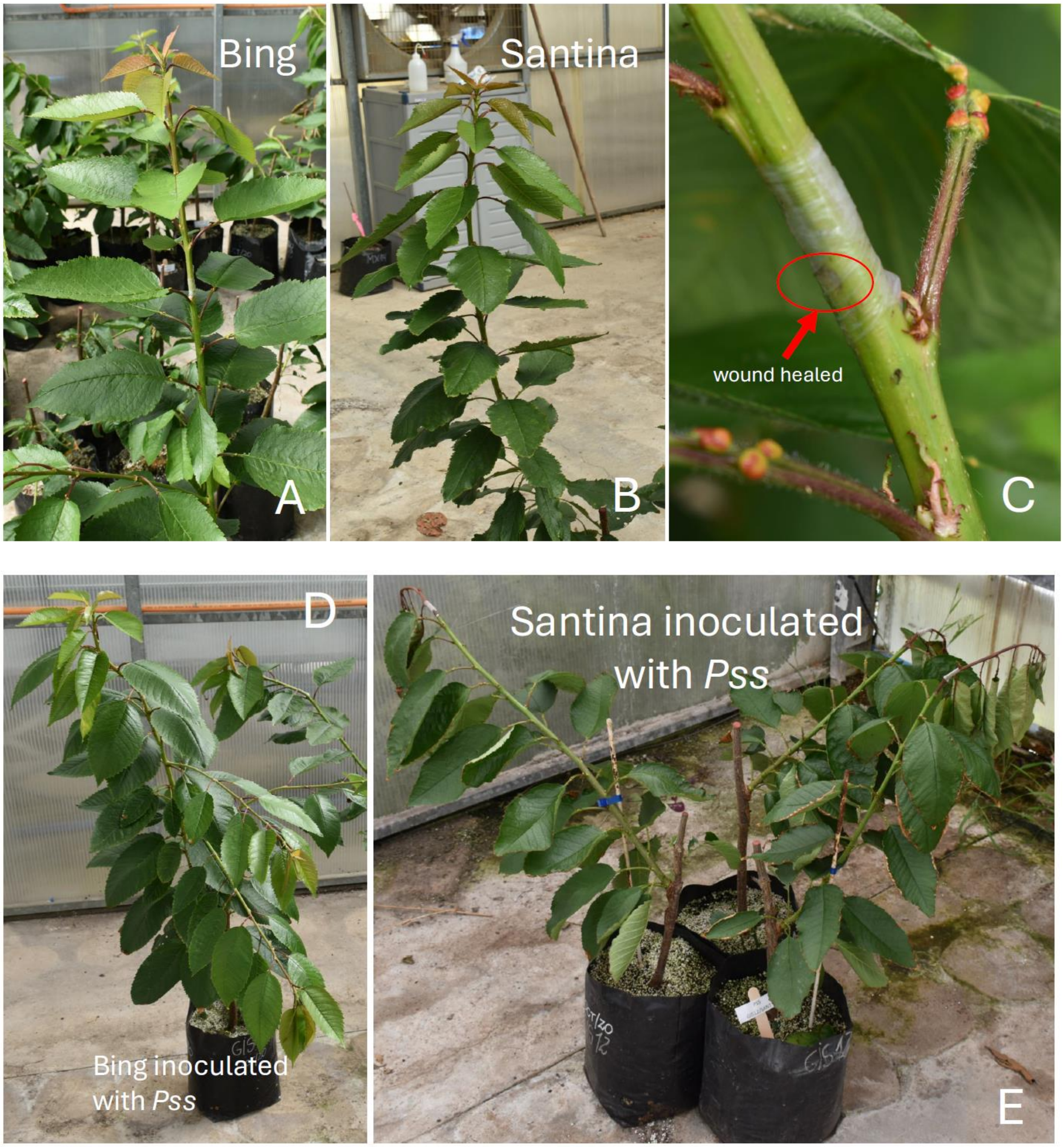

Supplemental data S5. *Pss* load quantification of *Prunus avium* samples inoculated with *Pss*A1M3 strain at 13 dpi near the inoculation site. DNA quantification of *Pss* and *Prunus avium* was performed in twig samples near the inoculation zone with mock as control or with *Pss*A1M3 strain at 10^8 CFU/mL. Mean PCR cycle number (Cq) value (performed in duplicate at least) and DNA concentrations are shown for each sample. *Pss* load presents the ratio between DNA quantification of *Pss* and *Prunus avium* in each biological replicate.

Inoculation	Variety	Sample	*Pss*	*P. Avium*	DNA ratio *Pss/P. avium*
			Pss ng/μL	*P. avium* ng/μL	(ng/ng)
Control	'Bing'	S1	0	6.1×10^{-1}	0
Control	'Bing'	S2	0	3.7×10^{-1}	0
Control	'Bing'	S3	1.4×10^{-6}	2.8	5×10^{-7}
Control	'Santina'	S1	0	1.2	0
Control	'Santina'	S2	0	1.6×10^{-1}	0
Control	'Santina'	S3	6.7×10^{-7}	4.9×10^{-1}	1.4×10^{-6}
Pss	'Bing'	S1	1.3×10^{-6}	4.3×10^{-1}	3×10^{-6}
Pss	'Bing'	S2	4.7×10^{-3}	8.7×10^{-1}	5.4×10^{-3}
Pss	'Bing'	S3	1.9×10^{-5}	6.7×10^{-2}	2.8×10^{-4}
Pss	'Santina'	S1	7.1×10^{-7}	5.2×10^{-1}	1.4×10^{-6}
Pss	'Santina'	S2	2×10^{-5}	2.1×10^{-1}	9.5×10^{-5}
Pss	'Santina'	S3	6×10^{-6}	1.1	5.5×10^{-6}
	C+ *Pss*		3.1	-	-
	C+ *P. avium*		-	5.3×10^{-1}	-
	C-		0	0	-

ND, not detected; -, not determined. C+ *Pss*, purified *Pss*A1M3 strain DNA; C+ *P. avium*, purified sweet cherry twig DNA. C-, sterile water.

Supplemental Data S6. Microbiological analysis for bacterial growth in sweet cherry plants shoots beyond 5 cm of inoculation site. Fluorescence emission was confirmed under a UV light.

Treatment	Variety	Sample	Bacterial growth	Fluorescence emission
Control	‘Bing’	S1	yes	no
Control	‘Bing’	S2	yes	no
Control	‘Bing’	S3	yes	no
Control	‘Santina’	S1	yes	yes
Control	‘Santina’	S2	yes	no
Control	‘Santina’	S3	yes	no
Pss	‘Bing’	S1	yes	no
Pss	‘Bing’	S2	no	no
Pss	‘Bing’	S3	no	no
Pss	‘Santina’	S1	yes	no
Pss	‘Santina’	S2	yes	no
Pss	‘Santina’	S3	yes	no

Supplemental data S7. PCR gel electrophoresis for *Pss* detection in sweet cherry shoots beyond 5 cm of the inoculation site. Lanes 1 and 2 represented replicates of the Santina sample exhibiting fluorescent emission, while c+ corresponded to the PssA1M3 positive control. The presence of *Pss*A1M3 was not detected below 5 cm from the inoculation site. The fluorescent bacteria likely belonged to other Pseudomonas

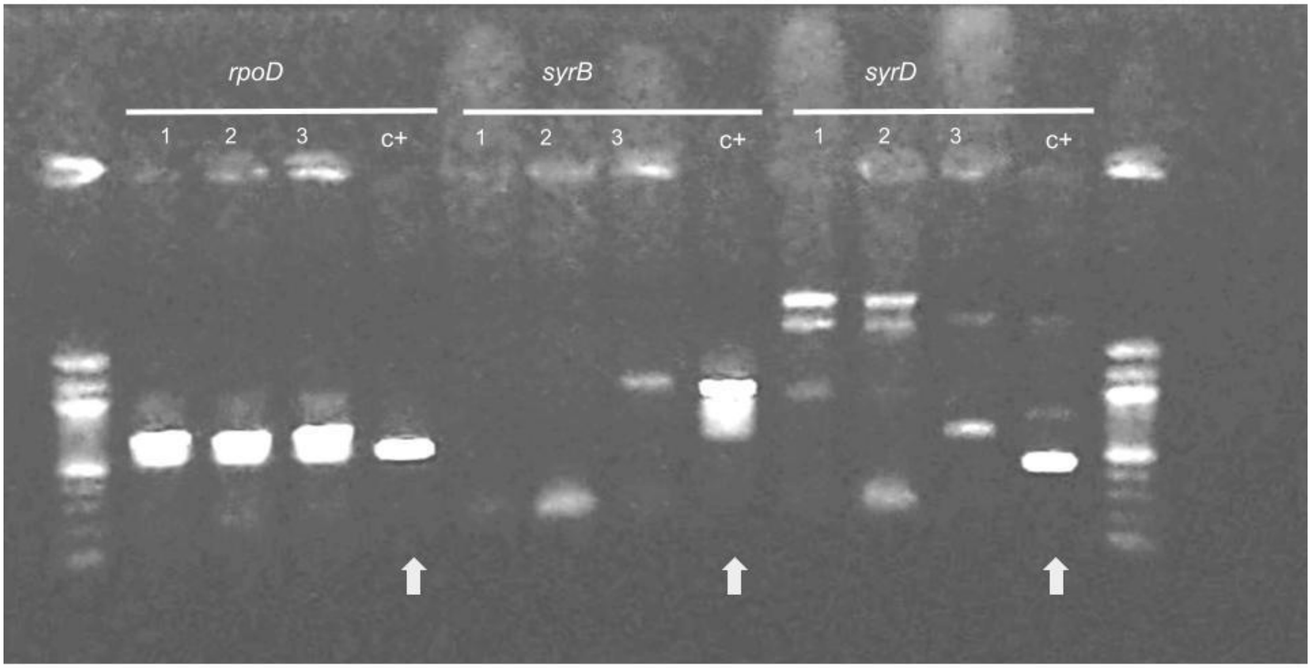

Supplemental data S8. Slopes analysis for whole plant Transpiration from 70 dpi to 90 dpi. Table shows the slope ± SE for the emtrends analysis. Different letters denote significant differences between varieties, inoculation, and irrigation treatments.

variety	irrigation	inoculation	slope	SE	df	lower.CL	upper.CL	
Santina	WW	control	11.05	1.083	328	8.92	13.19	a
Bing	WW	Pss	9.67	0.988	328	7.72	11.61	ab
Bing	WW	control	8.59	1.083	328	6.46	10.72	ab
Santina	WW	Pss	5.56	0.988	328	3.62	7.5	b
Bing	WD	control	-3.56	0.967	328	-5.46	-1.65	c
Santina	WD	control	-4.05	0.959	328	-5.94	-2.17	c
Santina	WD	Pss	-4.51	0.967	328	-6.42	-2.61	c
Bing	WD	Pss	-4.98	0.959	328	-6.86	-3.09	c

Degrees-of-freedom method: containment
Confidence level used: 0.95
P value adjustment: tukey method for comparing a family of 8 estimates
significance level used: alpha = 0.05
NOTE: If two or more means share the same grouping symbol,
then we cannot show them to be different.
But we also did not show them to be the same.

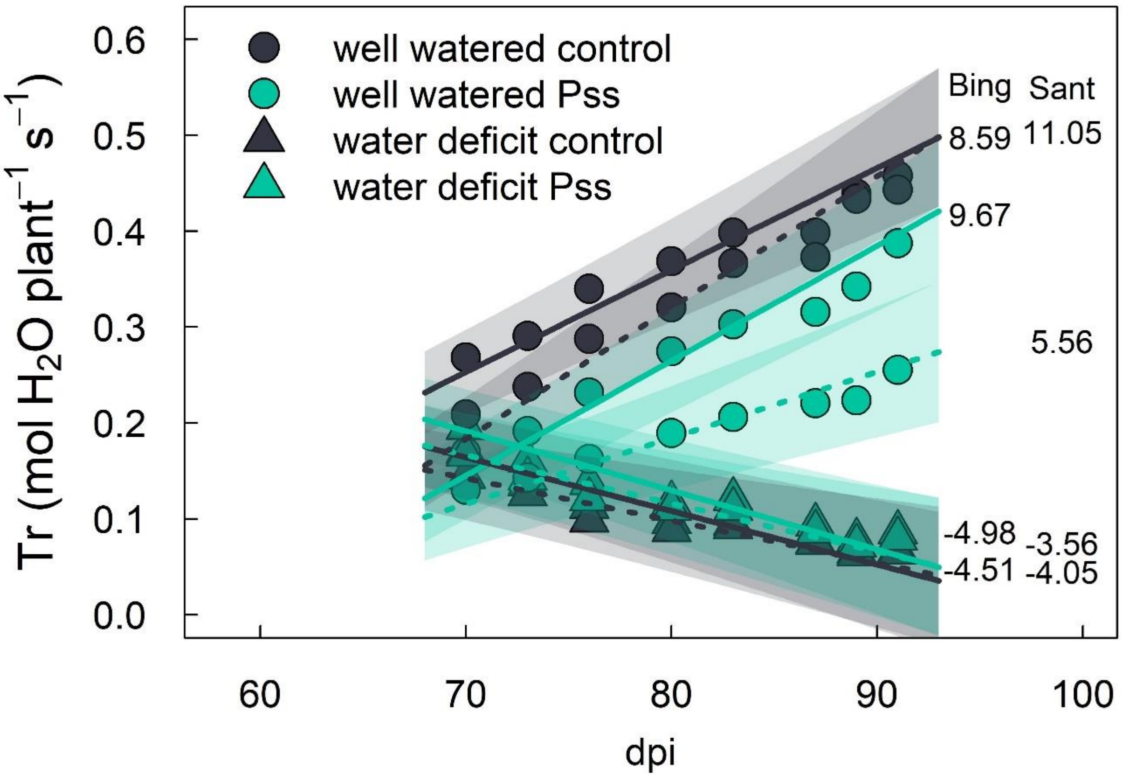

Supplemental data S9. *Pss* load quantification of *Prunus avium* leaves at 81 dpi. DNA quantification was performed on mature leaf samples taken from the vicinity of the inoculation zone on twigs, which had been inoculated with either a mock or the *Pss*A1M3 strain at 10^8 CFU/mL. Mean Cq value (performed in duplicate at least) and DNA concentration are shown for each sample. *Pss* load presents the ratio between DNA quantification of *Pss* and *Prunus avium* in each biological replicate.

Inoculation	Variety	Sample	*Pss*	*P. Avium*	DNA ratio *Pss/P. avium*
			ng/µL	ng/µL	(ng/ng)
Control	'Bing'	S1	0	$1.1x10^{-2}$	0
Control	'Bing'	S2	0	$3.6x10^{-2}$	0
Control	'Bing'	S3	0	$3.3x10^{-2}$	0
Control	'Bing'	S4	0	$2.1x10^{-2}$	0
Control	'Bing'	S5	0	$8.9x10^{-2}$	0
Control	'Bing'	S6	0	$7.4x10^{-2}$	0
Control	'Santina'	S1	0	$2x10^{-2}$	0
Control	'Santina'	S2	0	$1.8x10^{-2}$	0
Control	'Santina'	S3	0	$4.6x10^{-2}$	0
Control	'Santina'	S4	0	$2.3x10^{-1}$	0
Control	'Santina'	S5	0	$5.9x10^{-2}$	0
Control	'Santina'	S6	$2x10^{-6}$	$4.5x10^{-2}$	$4.4x10^{-5}$
Pss	'Bing'	S1	0	$3.8x^{-2}$	0
Pss	'Bing'	S2	0	$7.4x10^{-2}$	0
Pss	'Bing'	S3	0	$3x10^{-2}$	0
Pss	'Bing'	S4	0	$1.3x10^{-1}$	0
Pss	'Bing'	S5	0	$6.3x10^{-2}$	0
Pss	'Bing'	S6	0	$3.7x10^{-2}$	0
Pss	'Santina'	S1	0	$3.5x10^{-3}$	0
Pss	'Santina'	S2	0	$2.5x10^{-2}$	0
Pss	'Santina'	S3	$1.7x10^{-6}$	$2.8x10^{-2}$	$6.1x10^{-5}$
Pss	'Santina'	S4	0	$7.3x10^{-2}$	0
Pss	'Santina'	S5	0	$4x10^{-2}$	0
	C+ *Pss*		$1.1x10^{-2}$	-	-
	C+ *P. avium*		-	$4.6x10^{-1}$	-
	C-		0	0	-

ND, not detected; -, not determined. C+ *Pss*, purified *Pss*A1M3 strain DNA; C+ *P. avium*, purified sweet cherry leaf DNA. C-, sterile water.

Supplemental data S10. *Daily meteorological and agroclimatic data during the experiment in the summer season.* (**A**) Average daily air temperature (white) and degree days, GDD (red). The gray line indicates the thermal oscillation. The black arrows denote the beginning of irrigation treatments, and gray arrows denotes physiological measurements. (**B**) Average atmospheric water vapor pressure deficit, VPD (white) and reference evapotranspiration (light blue). The gray line indicates the VPD daily oscillation. Summer starts on December 22nd. 60 dpi corresponds to January 18th, 2022.

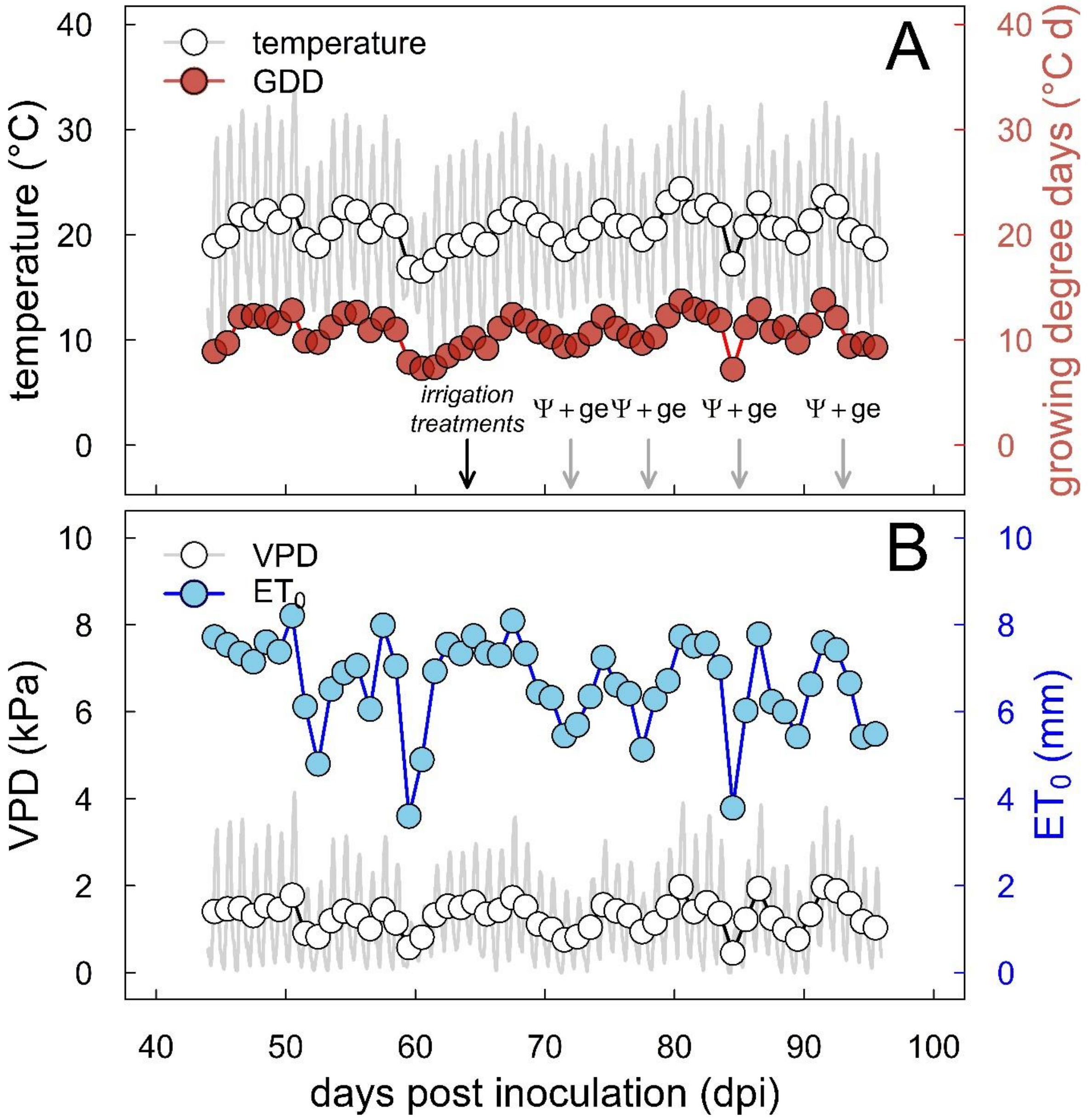

Supplement: Supplementary file 1 [file plants-13-01737-s001.zip › plants-2985893-supplementary.pdf]
